# Analysis of determinants of long-term efficacy of unboosted atazanavir-based regimens in the clinical setting

**DOI:** 10.1186/1758-2652-13-S4-P48

**Published:** 2010-11-08

**Authors:** S Bonora, A Calcagno, O Viganò, P Bigliano, S Rusconi, L Trentini, MC Tettoni, G Orofino, B Salassa, C Bramato, A D'Avolio, M Siccardi, E Colella, M Galli, G Di Perri

**Affiliations:** 1 University of Torino, Department of Infectious Diseases, Torino, Italy; 2 University of Milano, Luigi Sacco Hospital, Department of Infectious Diseases, Milano, Italy; 3 Ospedale Amedeo di Savoia, ASLTO2, Department of Infectious Diseases, Divisione, Torino, Italy; 4 University of Torino, Pharmacokinetics and Pharmacogenetics Laboratory, Torino, Italy

## Background

Switch to unboosted atazanavir (ATV 400 mg qd), although not licensed in Europe, is an attractive off-label option due to convenience and tolerability. However there is a substantial lack of data concerning efficacy of ATV 400 mg-based regimens outside the trial setting. Our aim was to perform a retrospective study of determinants of long term efficacy in 2 Italian large HIV clinics.

## Materials and methods

A retrospective analysis of virological (responder = last viral load < 50 copies/ml) and PK data of patients (pts) administered with ATV 400 mg QD + 2 N(Nt)RTIs for at least 3 months was performed. Genotypic Sensitivity Score (GSS) and ATV resistance associated mutations (RAMs) were calculated according to Stanford database using cumulative genotype. ATV Ctrough was measured by a validated HPLC method.

## Results

246 patients [65% male, mean age 47.5 years (±9), mean BMI 23.8 Kg/m^2^ (±3,4)] were considered. 40.7% and 6.6% were HCV- or HBV-coinfected, respectively, of whom 23 (9.3% of total) were cirrhotic. 32,9% and 17.9% of pts showed previous virological failure to NNRTIs and to PIs, respectively. Switch to ATV was mainly (48,4%) due to toxicity [dyslipidemia (21,5%) and gastrointestinal side effects (9,7%)] and to simplification (28,5%); last regimen was boosted PI-based in 178 patients (72.3%, 48% of whom ATV/r) and NNRTI-based in 24 (9.8%). At baseline CD4+ cell count was 428 cell/mm3 (±223) and 58,1% showed undetectable viral load. 212 (86.5%) patients had previous genotype available: backbone GSS was < 2 in 36,9% of patients and 6,7% had at least 1 ATV-RAM. Mean (±SD) follow-up was 120 weeks (± 64), and 235 (95.5%) pts were responders (74.4% still on treatment) while 11 (4.5%) showed a virological failure (3 showed selection of ATV-RAMs). ATV Ctrough (available in 84 patients) was higher in responders (median value 130 vs. 70 ng/ml), although this was not statistically significant (p>0.05). At multivariate analysis, GSS<2, ATV-RAMs ≥1 and self-reported non-adherence were associated with failure. ATV was stopped only in 4 patients (1.6%) for side effects.

## Conclusions

Over a mean follow up of more than 2 years, unboosted ATV showed high efficacy with good tolerability even in a clinical cohort including moderately experienced pts. However, due to the impact of GSS of backbone and presence of ATV-RAMs on the risk of failure, pts need to be accurately selected. The role of ATV plasma exposure deserves to be clarified on a larger sample size.

